# Supplementary material for: The causal relationship between the human gut microbiota and pyogenic arthritis: a Mendelian randomization study
Source: Front Cell Infect Microbiol. 2024 Nov 26;14:1452480. doi: 10.3389/fcimb.2024.1452480 (PMC11629706; doi:10.3389/fcimb.2024.1452480)
Supplement: Supplementary file 1 [file DataSheet1.zip › mendelian test/ebi-a-GCST90027610.csv_forest.pdf]

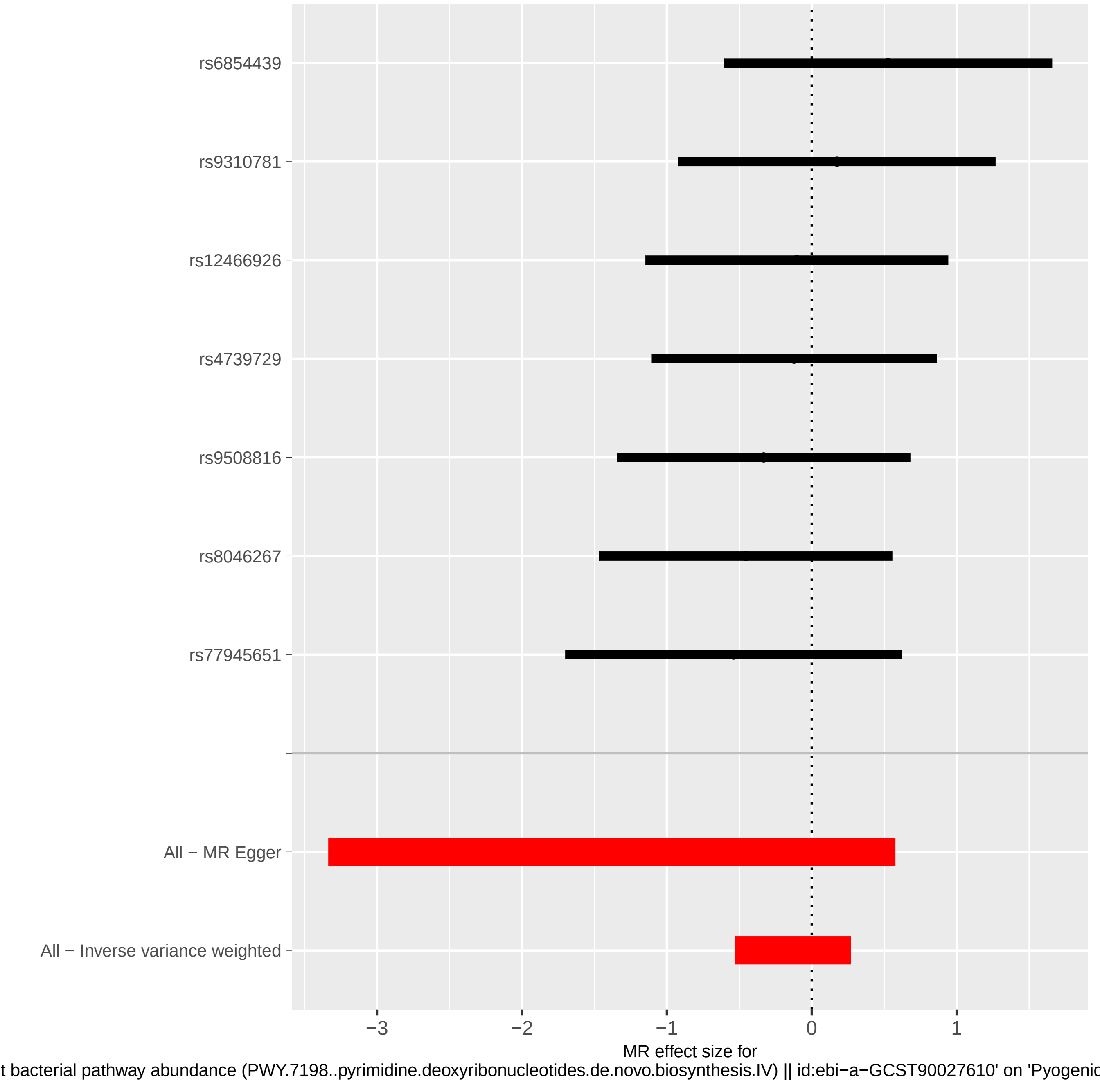

rs6854439
rs9310781
rs12466926
rs4739729
rs9508816
rs8046267
rs77945651
All − MR Egger
All − Inverse variance weighted
−3
−2
−1
0
1
MR effect size for
t bacterial pathway abundance (PWY.7198..pyrimidine.deoxyribonucleotides.de.novo.biosynthesis.IV) || id:ebi−a−GCST90027610' on 'Pyogenic
